# CT-based conventional radiomics and quantification of intratumoral heterogeneity for predicting benign and malignant renal lesions

**DOI:** 10.1186/s40644-024-00775-8

**Published:** 2024-10-02

**Authors:** Shuanbao Yu, Yang Yang, Zeyuan Wang, Haoke Zheng, Jinshan Cui, Yonghao Zhan, Junxiao Liu, Peng Li, Yafeng Fan, Wendong Jia, Meng Wang, Bo Chen, Jin Tao, Yuhong Li, Xuepei Zhang

**Affiliations:** 1 Department of Urology, The First Affiliated Hospital of Zhengzhou University, Zhengzhou, China; 2 Department of Information Management, The First Affiliated Hospital of Zhengzhou University, Zhengzhou, China; 3 Department of Radiology, The First Affiliated Hospital of Zhengzhou University, Zhengzhou, China; 4 Department of Urology, Tongliao Clinical College, Inner Mongolia Medical University, Tongliao, China

**Keywords:** Renal cell carcinoma, Benign renal lesion, Small renal lesion, Radiomics, Intratumoral heterogeneity, Computed tomography

## Abstract

**Background:**

With the increasing incidence of renal lesions, pretreatment differentiation between benign and malignant lesions is crucial for optimized management. This study aimed to develop a machine learning model utilizing radiomic features extracted from various regions of interest (ROIs), intratumoral ecological diversity features, and clinical factors to classify renal lesions.

**Methods:**

CT images (arterial phase) of 1,795 renal lesions with confirmed pathology from three hospital sites were split into development (1184, 66%) and test (611, 34%) cohorts by surgery date. Conventional radiomic features were extracted from eight ROIs of arterial phase images. Intratumoral ecological diversity features were derived from intratumoral subregions. The combined model incorporating these features with clinical factors was developed, and its performance was compared with radiologists’ interpretation.

**Results:**

Combining intratumoral and peritumoral radiomic features, along with ecological diversity features yielded the highest AUC of 0.929 among all combinations of features extracted from CT scans. After incorporating clinical factors into the features extracted from CT images, our combined model outperformed the interpretation of radiologists in the whole (AUC = 0.946 vs 0.823, *P* < 0.001) and small renal lesion (AUC = 0.935 vs 0.745, *P* < 0.001) test cohorts. Furthermore, the combined model exhibited favorable concordance and provided the highest net benefit across threshold probabilities exceeding 60%. In the whole and small renal lesion test cohorts, the AUCs for subgroups with predicted risk below or above 95% sensitivity and specificity cutoffs were 0.974 and 0.978, respectively.

**Conclusions:**

The combined model, incorporating intratumoral and peritumoral radiomic features, ecological diversity features, and clinical factors showed good performance for distinguishing benign from malignant renal lesions, surpassing radiologists’ diagnoses in both whole and small renal lesions. It has the potential to save patients from unnecessary invasive biopsies/surgeries and to enhance clinical decision-making.

**Supplementary Information:**

The online version contains supplementary material available at 10.1186/s40644-024-00775-8.

## Introduction

In 2020, renal cell carcinoma (RCC) accounted for 431,288 new cases and 179,368 deaths worldwide [1]. The incidence of RCC has been on the rise over the past two decades, partly due to the widespread adoption of ultrasonography and cross-sectional imaging [2, 3]. Deciding whether to perform resection of cystic or solid renal lesions is often based on clinical imaging, without definitive histologic diagnosis [4, 5]. Studies have revealed that about one-quarter of surgically removed renal lesions were reported to be benign, and the percentages of benign lesions increased as the diameter of lesion decreased [6, 7]. This poses challenges in determining the appropriate method and necessity of treatment for all suspicious lesions, particularly for small renal lesions (SRL, defined as ≤ 4.0 cm in diameter). Improved diagnosis and differentiation of SRLs has been identified as a key research focus in RCC by an international research priority setting initiative [8].

Traditionally, CT is the routine modality used in clinical practice to characterize renal lesions. However, its sensitivity and specificity are limited in distinguishing between benign indolent lesions and aggressive malignant renal lesions [9, 10]. A reliable and accurate method for diagnosing of renal lesions, especially small ones, is desired to reduce the need for biopsy and resection of benign entities. Radiomics comprises a diverse set of techniques designed to convert medical images into quantitative and high-dimensional data, allowing for the identification of complex patterns not recognized by the human eye [11]. The application of radiomics features has been proven valuable in the differentiation of renal lesions. However, most studies on this topic were limited by small sample sizes, typically ranging from 84 to 252 participants [12–18]. Moreover, these studies primarily focused on intratumoral radiomics, overlooking the significance of peritumoral radiomics and intratumoral heterogeneity (ITH), which could provide a unique viewpoint in tumor interpretation [19, 20]. Additionally, many of these studies focused on common pathological types [12–18], which was incongruent with real-world clinical workflow, as it assumed that uncommon pathological types of renal lesions are already excluded. While a few studies with larger sample sizes (> 500) were reported, and some included uncommon and unclassified RCC subtypes, the benign category still only include oncocytoma and angiomyolipoma (AML) [21–23]. Therefore, larger and more diverse patient cohorts are essential to advance algorithm development.

Our study cohort is sourced from a database of patients who underwent partial nephrectomy, encompassing a diverse range of pathological types, with most lesions being small (≤ 4.0 cm). The purpose of our study was to develop a machine learning model based on intratumoral and peritumoral radiomic features, intratumoral ecological diversity features, and clinical factors from this large database to predict benign or malignant renal lesions.

## Patients and methods

### Patients and clinical data

**Fig. 1:**
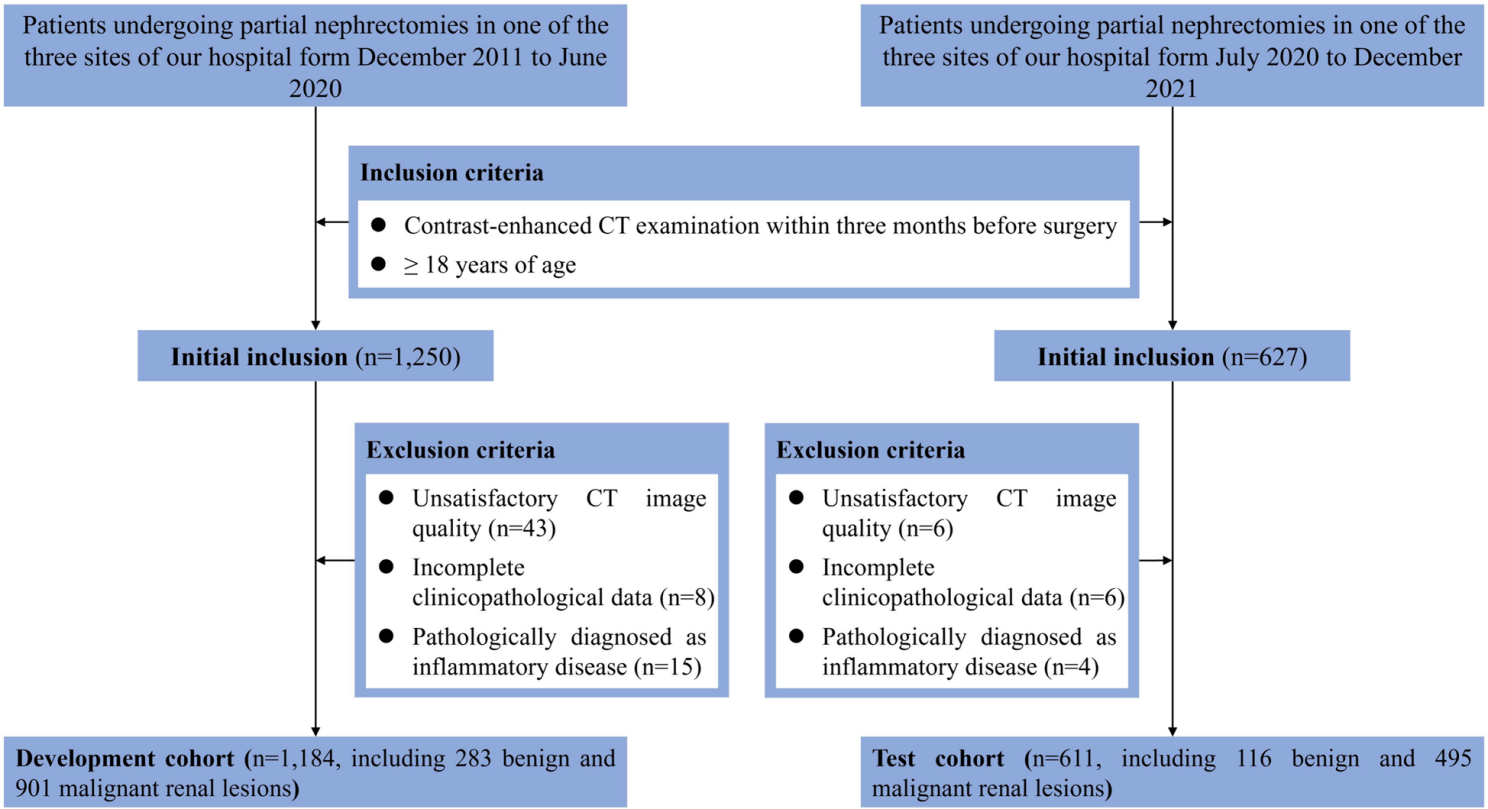
Flowchart of study participants inclusion

The retrospective study was conducted in accordance with Declaration of Helsinki, and approved by the Institutional Ethics Review Board with waivers for informed consent. From December 2011 to December 2021, a total of 1,877 patients underwent partial nephrectomies and received contrast-enhanced CT examinations were included from three different sites of our hospital. Out of these, 1,795 (95.6%) patients were included in the final analysis according to the inclusion and exclusion criteria shown in Fig. 1. The patients were divided into development and test cohorts based on the date of surgery. The development cohort consisted of 1,184 (66.0%) patients treated between December 2011 and June 2020, while the test cohort included 611 (34.0%) patients treated from July 2020 to December 2021 (Fig. 1).

Patient clinical and pathological data was collected and reviewed, including: (1) demographic and clinical characteristics, namely, gender, date of birth, laterality and size of lesion, date of surgery; (2) CT data, namely, arterial (1795 cases, 100.0%), non-contrast (1502, 83.7%), and venous (1,478, 82.3%) phase CT imaging, date of CT imaging, and CT reported results; (3) pathology data, namely, date of pathology report, and pathological conclusion. The pathological diagnoses were retrieved from the pathological conclusions, which were reported by a pathologist with a minimum of 3 years in genitourinary pathology and reviewed by a specialist with over 10 years’ experience.

### CT examination and radiologic evaluation

All CT scans were performed with one of the following scanners: Phillips 256 iCT, GE Discovery CT750 HD scanner, or GE Revolution CT. The CT scanning parameters were shown in Appendix S1. The CT image was interpreted by one radiologist with at least 3 years of experience in abdominal radiology and reviewed by another radiologist with at least 10 years of experience. CT reported results for renal lesions were classified as malignant, equivocal, and benign based on CT reports, by individuals blinded to the pathological results.

### ROI definition and radiomic analysis

**Fig. 2:**
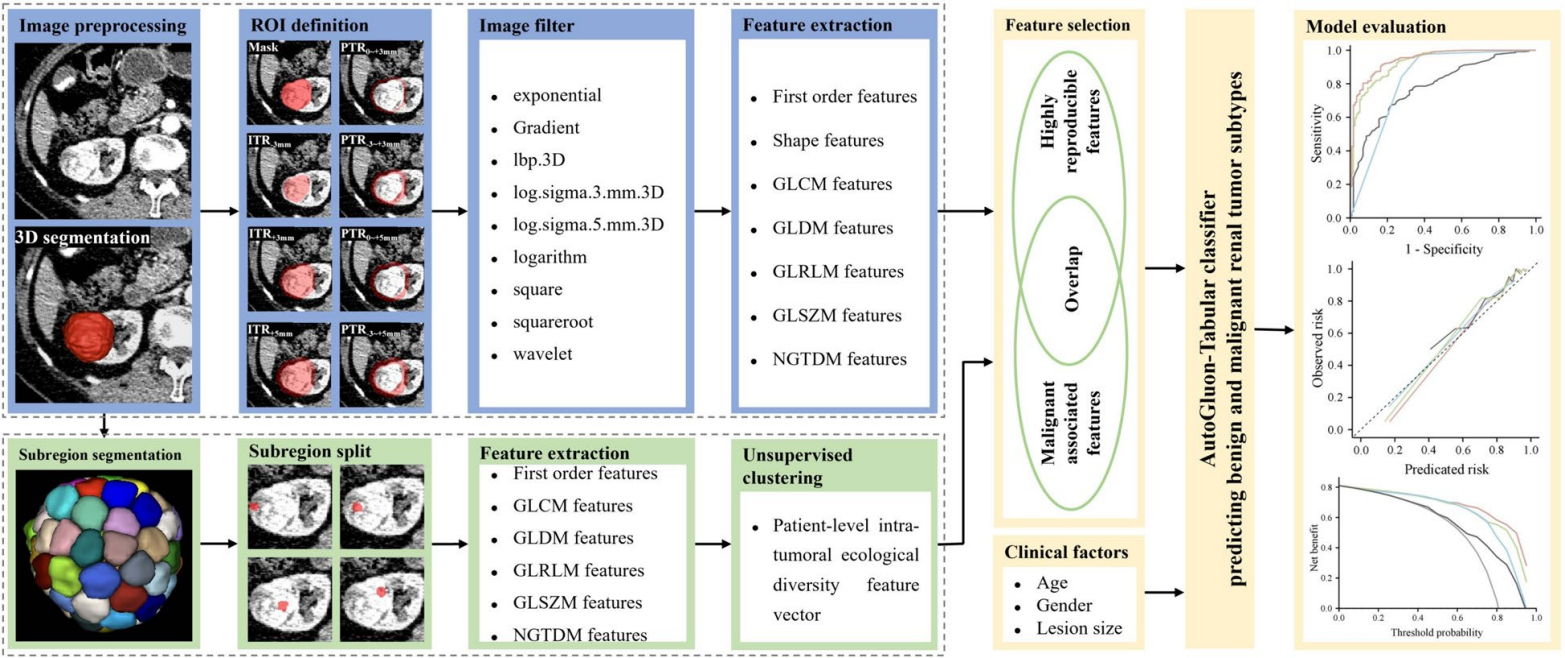
Workflow of developing the model based on intratumoral radiomics, peritumoral radiomics, intratumoral heterogeneity, and clinical factors to distinguish benign and malignant renal lesions

The radiomics workflow is depicted in Fig. 2. CT images segmentation, definition of regions of interest (ROIs), registration, and feature extraction were detailed in Appendix S2. All eight ROIs, namely, intratumoral region (ITR), ITR with 3 mm shrink (ITR_-3 mm_), ITR with 3 mm (ITR_+3 mm_) and 5 mm (ITR_+5 mm_) expansion, peritumoral regions (PTRs) of 3 mm (PTR_0~+3 mm_) and 5 mm (PTR_0~+5 mm_) around the tumors, as well as 6 mm (PTR_-3~+3 mm_) and 8 mm (PTR_-3~+5 mm_) crossing tumor border, were used to extract radiomic features from arterial phase images. A total of 14,248 (1,781 × 8) radiomic features were extracted from each renal lesion of arterial phase image. For non-contrast and venous phase images, only ITR were used to extract radiomic features. The radiomics features extracted in this study followed an internationally standardized and reproducible approach, adhering to the definitions outlined by the Imaging Biomarker Standardized Initiative [24–26].

### Intratumoral subregions segmentation and heterogeneity analysis

To objectively segment the ITR of the arterial phase into subregions, a simple linear inactive clustering method was employed with a predefined segment number (*n* = 100) (Fig. 2) [20, 27]. Radiomic features (first order, GLCM, GLDM, GLRLM, GLSZM and NGTDM) were extracted from each tumor segment. Subsequently, an unsupervised clustering was conducted using Gaussian mixture model based on each feature across all segments of a patient. The optimal number of clusters, indicative of tumor ecosystem diversity, was determined using Bayesian information criteria, ranging from 1 to 5 (Fig. 2). For example, the first order feature entropy specifies the uncertainty or randomness in the image values, which measures the average amount information required to encode the image values. The intratumoral ecological diversity feature of entropy specifies the complexity level of intratumoral heterogeneity when we analyze a tumor through entropy. Finally, each patient has an intratumoral ecological diversity feature vector (93 features) for further analysis, as proposed by Shi et al. [20].

### Model construction and validation

The feature selection process in the development cohort consisted of two steps: unstable features with intraclass correlation coefficient (ICC) less than 0.85 were excluded; and features that did not exhibit significant differences between benign and malignant lesions were removed using Student’s t-test. The radiomic signatures and ITH index was constructed by a stacked ensemble model (AutoGluon-Tabular classifier, Version 0.8.2) based on the stable and significant features. To optimize the hyperparameters, a five-fold stratified cross-validation within the development cohort was employed. AutoGluon was run with parameters set to “eval_metric = ’roc_auc’, and presets = ’best_quality’”, while all other parameters were left at their default settings.

Simultaneously, we constructed radiomic signatures and ITH index using the LASSO algorithm. AutoGluon outperformed LASSO in the cross-validation on development cohort and in the test cohort (Fig. S1), thus was chosen as classifier for constructing the combined model based on the combination of intratumoral radiomics features, peritumoral radiomic features, intratumoral ecological diversity features, and clinical factors. Feature importance score was generated using the “feature_importance” function of AutoGluon-Tabular classifier with default parameters. A list of features with a positive feature importance score and a *P* < 0.05 was used to predict benign and malignant renal lesions.

To evaluate the diagnostic ability of clinical factors and CT reported results, they were separately integrated into the Logistic Regression (LR) algorithm. Additionally, the predicted risk by the combined model was fused with the CT reported results to investigate whether it enhances the radiologists’ diagnostic performance.

### Statistical analysis

We presented continuous variables as median (range) and categorical variables as frequency (percentage). Continuous data were analyzed using Student's t-test, while categorical data using the χ^2^ test. The performance of models was evaluated using the area under the receiver operating characteristics (ROC) curve (AUC). The 95% confidence interval (CI) for the AUC and comparisons between AUCs were determined using the method of DeLong et al. [28]. Model performance was also examined using calibration plots, with calibration assessed by grouping cases in the test cohort into deciles and comparing the mean of predicted probabilities with observed proportions. The deviation of calibration plots from the 45° line was assessed using the sum squares of the residuals (SSR). The clinical utility of the models was evaluated using decision-curve analysis [29]. To further assess the clinical utility of the radiomics model, we sought to identify optimal cutoff values for different performance metrics. The cutoff values corresponding to 99% sensitivity (0.330 and 0.360), 95% sensitivity (0.593 and 0.590), 95% specificity (0.862 and 0.880), and 99% specificity (0.940 and 0.865) were calculated for the combined model in both the whole and SRL test cohorts.

## Results

### Clinicopathological characteristics

A total of 1,795 patients with confirmed renal lesion on contrast-enhanced CT imaging underwent partial nephrectomies and received definitive pathological diagnoses. The median age of the entire cohort was 54 years, with 61.5% (*n* = 1,104) being male and 63.4% (*n* = 1,138) having SRL (Table 1). Final pathology diagnosed 1,396 (77.8%) patients with RCC, including clear cell (85.3%), papillary (3.7%), chromophobe (5.6%), MiT Family translocation (2.3%), uncommon (1.1%), and unclassified (2.0%) RCC. The remaining 399 (22.2%) patients had AML (76.9%), oncocytoma (4.5%), benign cyst (13.8%), and uncommon benign renal tumor (4.8%) (Table 1).

**Table 1:** Patient clinical characteristics and histologic types

Parameters	Benign (*n* = 399)	Malignant (*n* = 1396)	*P* value
Age, median (range, years)	50 (18–84)	56 (18–86)	< 0.001
Gender, n (%)			< 0.001
Female	273 (68.4)	418 (29.9)	
Male	126 (31.6)	978 (70.1)	
Laterality, n (%)			0.480
Left	182 (45.6)	667 (47.8)	
Right	217 (54.4)	729 (52.2)	
Lesion size, median (range, cm)	3.9 (0.9–19.3)	3.5 (0.9–13.9)	< 0.001
CT reported results			< 0.001
Malignant	102 (25.6)	1146 (82.1)	
Equivocal	47 (11.8)	211 (15.1)	
Benign	250 (62.7)	39 (2.8)	
Lesion subtype, n (%)			-
Clear cell RCC	-	1191 (85.3)	
Papillary RCC	-	51 (3.7)	
Chromophobe RCC	-	78 (5.6)	
MiT Family translocation carcinomas	-	32 (2.3)	
Other malignant renal tumor	-	16 (1.1)	
Unclassified RCC	-	28 (2.0)	
Angiomyolipoma	307 (76.9)	-	
Oncocytoma	18 (4.5)	-	
Cyst	55 (13.8)	-	
Other benign renal tumor	19 (4.8)	-	

Other malignant renal tumors included 4 cases of multilocular cystic RCC, 4 cases of sarcomas, 3 cases of clear cell papillary RCC, 2 cases of well-differentiated neuroendocrine tumors, 1 case of succinate dehydrogenase deficient RCC, 1 case of collecting duct carcinoma, and 1 case of malignant perivascular epithelioid cell tumor. Other benign renal tumors included 6 cases of adult cystic nephroma, 4 cases of juxtaglomerular cell tumor, 3 cases of metanephric adenoma, 2 cases of papillary adenoma, 2 cases of inflammatory myofibroblastic tumors, 1 case of mixed epithelial and stromal tumor, and 1 case of schwannoma

*RCC* Renal cell carcinoma

**Fig. 3:**
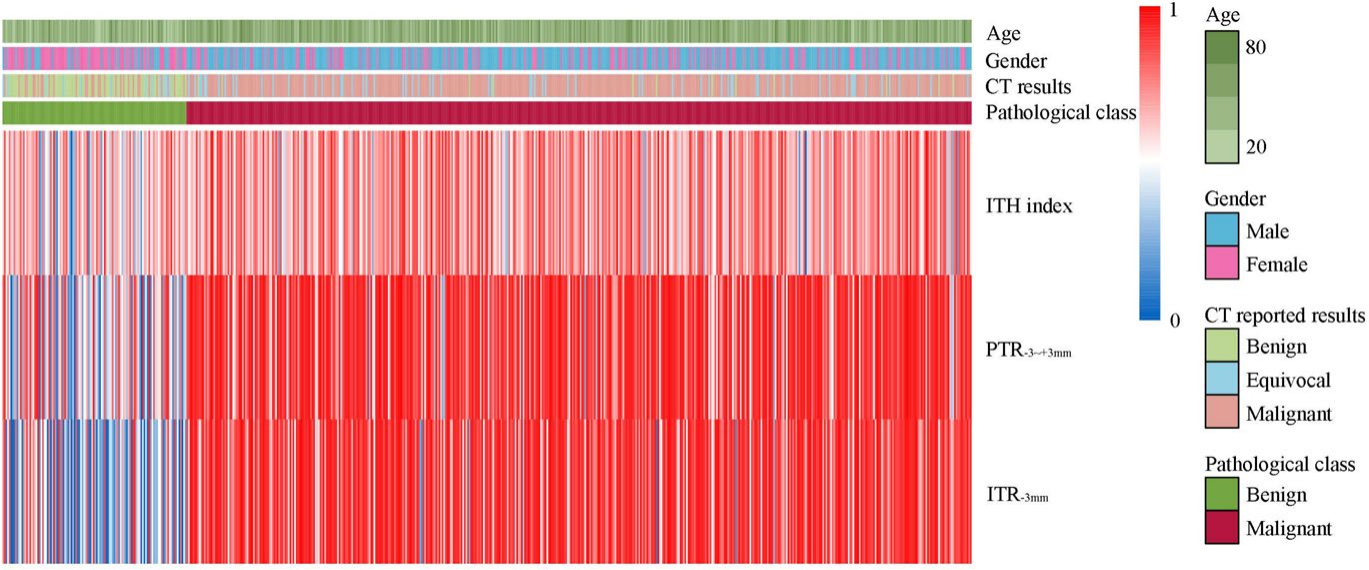
Heat map shows the association of pathological class with clinical factors (age, gender, CT reported results) and CT features (intratumoral heterogeneity index, peritumoral radiomics score, intratumoral radiomics score) in the test cohort. ITH: intratumoral heterogeneity; PTR_-3~+3 mm_: peritumoral regions of 6 mm crossing tumor border; ITR_-3 mm_: intratumoral region with 3 mm shrink

**Fig. 4:**
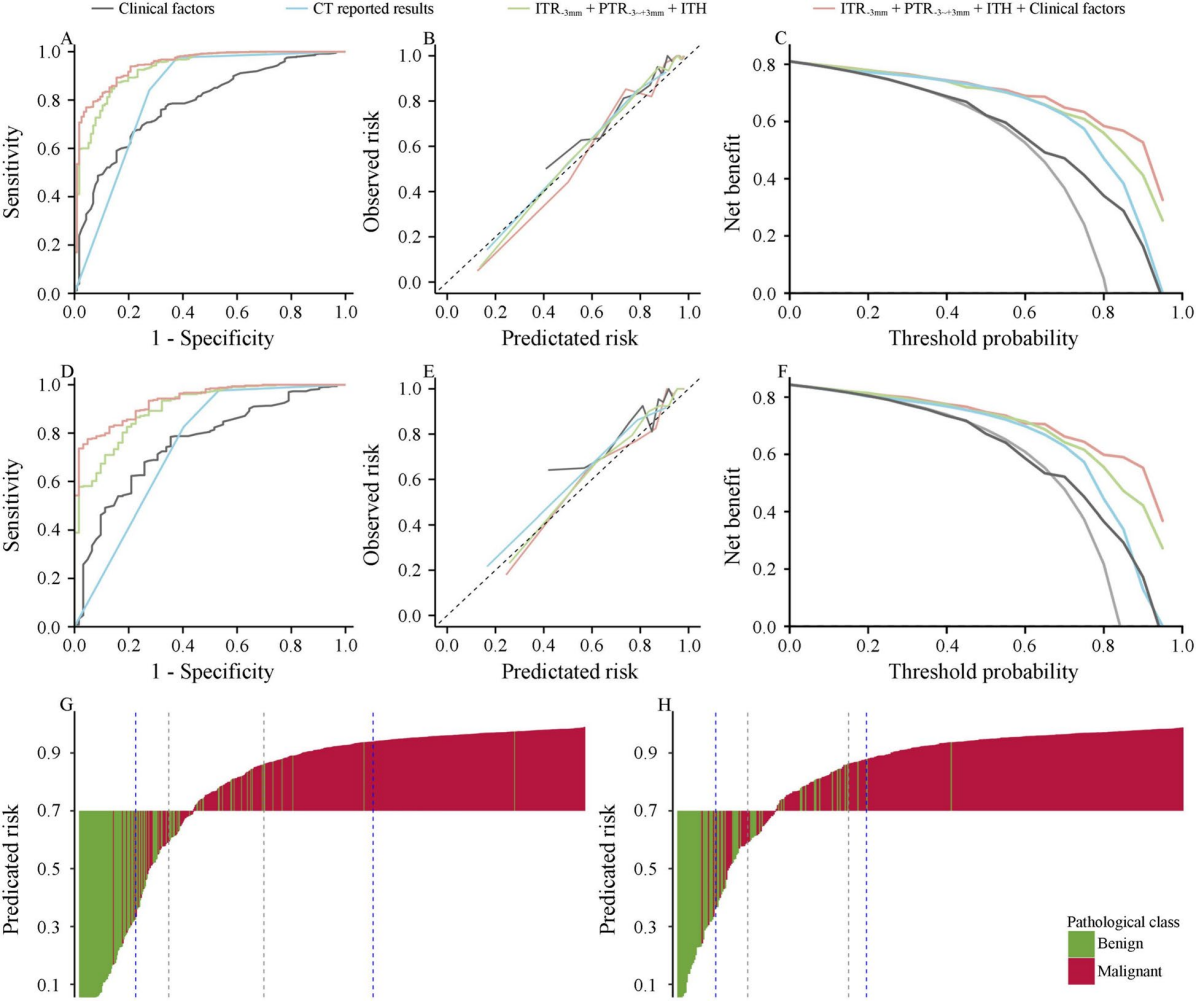
The performance of the clinical factors, CT reported results, radiomics-ITH model, and combined model for differentiation of benign from malignant renal lesions in the whole test cohort and in the test cohort with small renal lesion. ROC curves in the whole (**A**) and small renal lesion (**D**) test cohort; Calibration plot of observed vs predicated risk of malignant renal lesions in the whole (**B**) and small renal lesion (**E**) test cohort; Decision curve analysis for predicting malignant renal lesions in the whole (**C**) and small renal lesion (**F**) test cohort; Waterfall plot for predicated risk by the combined model in the whole (**G**) and small renal lesion (**H**) test cohort. The dashed lines from left to right indicates the cutoff values for 99% sensitivity, 95% sensitivity, 95% specificity, 99% specificity, respectively in the panel G and H. ITH: intratumoral heterogeneity; ROC: receive operating characteristic curves

In univariate analysis, age, gender, size of lesion, and CT reported results were significant variables for distinguishing benign and malignant renal lesions (each *P* < 0.001, Table 1). Multivariate LR was then performed to separately combine clinical factors and CT reported results. The clinical factors alone had an AUC of 0.784 (95% CI: 0.740–0.828), and the CT reported results alone yield an AUC of 0.823 (95% CI: 0.777–0.870) in the test cohort (Figs. 3 and 4A).

### Feature selection and construction of radiomic signatures and ITH index

Of the 14,248 radiomic features extracted from the eight ROIs in the arterial phase images, 9,963 (69.9%) with high stability (interobserver and intraobserver ICC ≥ 0.85) were initially selected. Next, 3,736 (37.5%) significant features were selected by Student's t-test (*P* < 0.001), including 388 (10.4%), 343 (9.2%), 455 (12.2%), 434 (11.6%), 535 (14.3%), 564 (15.1%), 503 (13.5%)and 514 (13.8%) radiomic features from the ITR, ITR_-3 mm_, ITR_+3 mm_, ITR_+5 mm_, PTR_0~+3 mm_, PTR_0~+5 mm_, PTR_-3~+3 mm_, and PTR_-3~+5 mm_ of arterial phase images, respectively. For non-contrast and venous phase images, 259 and 430 stable and significant features were selected, respectively. For ITH analysis, six stable and significant features were selected.

The AUC values for ten radiomic signatures, which include eight based on the arterial phase, one based on the non-contrast phase, and one based on the venous phase, ranged between 0.815 and 0.891 in the cross-validation, and between 0.757 and 0.889 in the test cohort (Fig. S1). Among these signatures, the radiomic signatures derived from ITR_-3 mm_ (AUC = 0.889, 95%CI: 0.852–0.926) and PTR_-3~+3 mm_ (AUC = 0.849, 95%CI: 0.807–0.890) exhibited the highest performance in the test cohort for intratumoral and peritumoral radiomic signatures, respectively. The AUC value for ITH index was 0.665 (95%CI: 0.609–0.722) in the test cohort (Fig. 3 and S1).

### Development and performance of the combined model

To enhance predictive accuracy, we developed models combining intratumoral and peritumoral radiomic features, as well as intratumoral ecological diversity features. The combination of radiomic features from ITR_-3 mm_ and PTR_-3~+3 mm_, along with ecological diversity features yielded the highest AUC of 0.929 (95% CI: 0.904–0.955) among all combinations of features extracted from CT scans (Fig. 4A and Table S1). The radiomics and ITH model included a total of 43 features, comprising of 19 radiomic features from ITR_-3 mm_, 23 features from PTR_-3~+3 mm_, and one ecological diversity feature (Table S2).

**Fig. 5:**
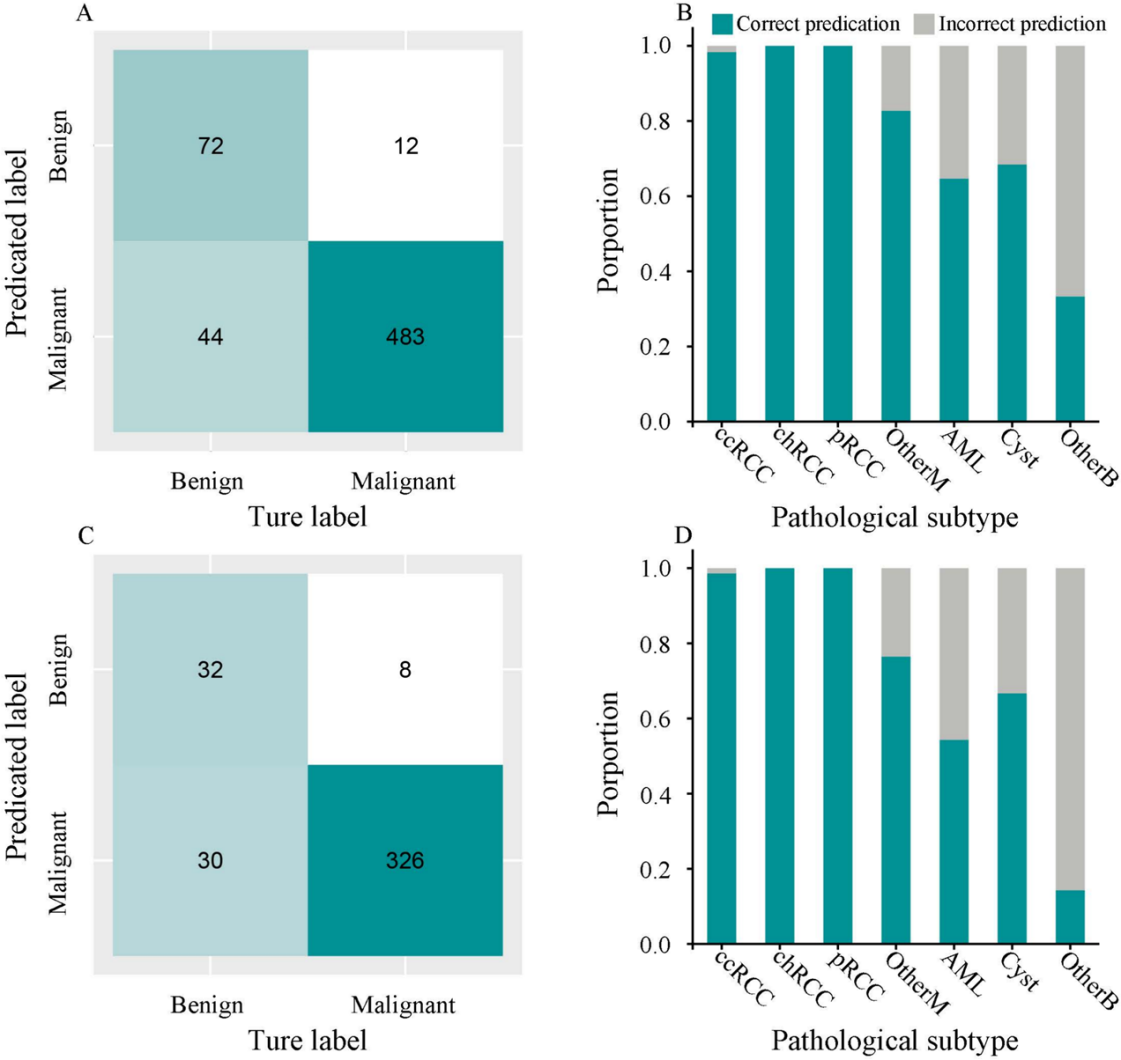
The performance of the combined model in the whole test cohort and in the test cohort with small renal lesion. Confusion matrices for the whole (**A**) and small renal lesion (**C**) test cohorts; The proportion of correct predictions by pathological subtype for the whole (**B**) and small renal lesion (**D**) test cohort

Using the AutoGluon-Tabular classifier, the addition of clinical factors to the features extracted from CT images improved the AUC to 0.946 (95%CI: 0.925–0.968) (Fig. 4A) in the test cohort. The feature importance for the combined model ranged from 0.0014 to 0.0380 (Table S2). The confusion matrix showed that most cases (555/611, 90.8%) were correctly predicted by the combined model (Fig. 5A). Pathological subtype analysis showed that the proportions of correct prediction for clear cell RCC, papillary RCC and chromophobe RCC were high (Fig. 5B). To assess whether the combined model could enhance the radiologists’ diagnostic performance, the fusion of predicated risk by the combined model and CT results demonstrated superior performance in both the whole test cohort (AUC = 0.954, 95%CI: 0.933–0.975) and SRL test cohort (AUC = 0.941, 95%CI: 0.914–0.968), significantly outperforming the CT results alone (*P* < 0.001, Fig. S2).

**Fig. 6:**
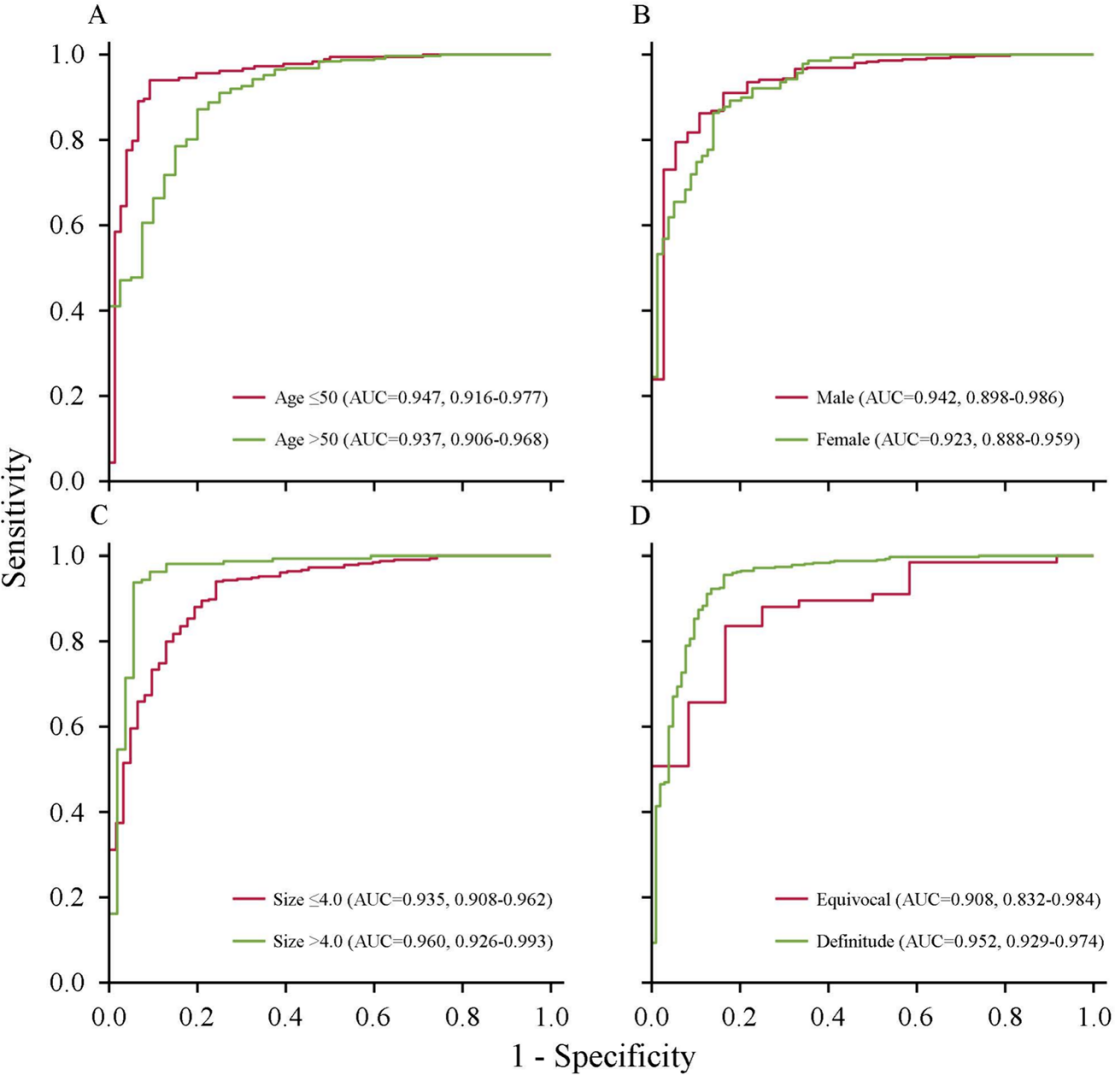
ROC curves of the combined model based on the clinical factors in the test cohort. Age (**A**); Gender (**B**); Lesion size (**C**); Category of CT reported results (**D**). ROC: receive operating characteristic curves

The test cohort was divided into subgroups by clinicopathological characteristics to assess the performance stability of the combined model (Fig. 6). The results showed that the combined model achieved relatively stable performance in subgroups by gender, with an AUC of 0.942 for males and 0.923 for females (Fig. 6B). However, the model’s performance was relatively weak in subgroups with age > 50 years (AUC = 0.937) (Fig. 6A), lesion size ≤ 4.0 cm (AUC = 0.935) (Fig. 6C), and CT reports indicating equivocal results (AUC = 0.908) (Fig. 6D).

A sensitivity analysis was conducted for patients with SRL. Of these patients, 215 (18.9%) had benign renal lesions and 923 (81.1%) had carcinoma on final pathology. The radiomics and ITH model demonstrated the ability to differentiate benign from malignant renal lesions in this subgroup, with an AUC of 0.904 (95% CI: 0.8.866–0943) (Fig. 4D). The addition of clinical factors to the features extracted from CT scans significantly improved the predictive performance (AUC = 0.935, 95% CI: 0.908–0.962), which was higher than the clinical factors (AUC = 0.756, *P* < 0.001), and CT reported results (AUC = 0.745, *P* < 0.001) in the SRL test cohort (Fig. 4D).

### Clinical impact of predictive models

The calibration plot of predicated risk against observed proportion of malignant renal lesions indicated favorable concordance for the combined model in the whole (SSR = 0.034) and SRL (SSR = 0.024) test cohorts (Fig. 4B and E). The decision curve analysis showed that the combined model demonstrated a higher net benefit than the other three predictions when the threshold probabilities exceeded 60% for both the whole and SRL test cohort (Fig. 4C and F).

The AUC for the subgroup with a predicated risk below the cutoff value of 99% sensitivity or above the cutoff value of 99% specificity were 0.987 (95% CI: 0.966–1.000) and 0.988 (95% CI: 0.959–1.000) in the whole and SRL test cohorts, respectively. Similarly, the AUC for the subgroup with a predicated risk below or above the cutoff value of 95% sensitivity or specificity were 0.974 (95% CI: 0.954–0.994) and 0.978 (95% CI: 0.959–0.997) in the whole and SRL test cohorts, respectively (Fig. 4G and H). Conversely, the AUC for subgroups with a predicated risk falling between the cutoff values of 95% sensitivity and specificity was 0.605 (95%CI: 0.468–0.741) and 0.451 (95%CI: 0.289–0.614) in the whole and SRL test cohorts, respectively. Hence, when the predicated probability is below or above the cutoff value of 95% sensitivity or specificity, the combined model provides doctors with greater confidence in clinical use. However, when the predicated risk falls between the cutoff values of 95% sensitivity and 95% specificity, doctors need to consider integrating other marker to make decisions.

## Discussion

Accurate differentiation of benign from malignant renal lesions poses a significant challenge for clinicians and is essential for optimizing patient management, particular for SRLs. In this study, we developed a machine learning model that leverages intratumoral and peritumoral radiomic features, intratumoral ecological diversity features, as well as clinical factors for pretreatment differentiate between benign and malignant renal lesions. Our combined model demonstrated a robust discriminative capability in distinguishing benign from malignant renal lesions, outperforming the interpretation of radiologists in the whole and SRL test cohorts.

In assessing the performance of radiomic signatures using features extracted from different CT phases, our study found that algorithms based on the features from arterial phase CT images demonstrated the highest diagnostic accuracy in renal lesion differentiation, consistent with findings by Tanaka et al. [16]. Moreover, adding features extracted from the non-contrast and venous phases (AUC = 0.909) to those extracted from ITR_-3 mm_ and PTR_-3~+3 mm_ of the arterial phase (AUC = 0.917) didn’t enhance the model’s performance (Table S1). Therefore, we chose to focus on the arterial phase for further analysis. Several previous studies have leveraged radiomic features to develop models for predicting benign and malignant renal lesions. These radiomics-based models achieved an AUC ranging from 0.790 to 0.915 [12–14, 22, 23, 30]. In parallel, several studies have used deep learning features to create models for differentiation benign from malignant renal lesions [16, 21, 31], with these models achieving an AUC of 0.730–0.933 in the test cohorts. In our research, we constructed radiomic signatures based on eight ROIs of arterial phase images, achieving AUCs of 0.811–0.889 in the test cohort, which are comparable to the previous studies. The radiomic signatures of ITR_-3 mm_ and PTR_-3~+3 mm_ had the highest performance among the intratumoral and peritumoral radiomic signatures, respectively. Furthermore, a study by Liu et al. highlighted that the radiomics signature of PTR_-3~+3 mm_ exhibited the highest accuracy in predicting of prognosis for clinical stage I solid lung adenocarcinoma [32]. These findings indicate that the inner and outer regions of tumor may provide different clinical insights.

Among all combinations of features extracted from CT scans, the pairing of radiomic features from ITR_-3 mm_ and PTR_-3~+3 mm_, in conjunction with ecological diversity features yielded the highest AUC, which was higher than the radiomic signature of ITR_-3 mm_. This finding suggests that the peritumoral radiomics and ITH are helpful to improve the performance of the intratumoral radiomics in distinguishing benign from malignant renal lesions. Despite peritumoral radiomics and ITH are known to correlate with tumor phenotypes [20, 32], the role of peritumoral radiomics and ITH in distinguishing of renal lesions has not been fully assessed. During tumor progression, the invasion of tumor into surrounding normal tissues at the cellular level may manifest as changes in tissue morphology. And ITH is influenced by both the diverse composition of cell populations and their uneven distributions within the tumor. These could provide a reasonable explanation for the enhanced diagnostic accuracy of radiomics models, attributed to peritumoral radiomics and ITH.

Until now, studies exploring the addition of clinical factors to the radiomics signature for deafferenting renal lesions have been rare. Several studies have explored the association between clinical predictors and the malignancy of renal lesions [21, 22, 33–35]. These studies consistently found that female sex was predominantly associated with benign pathologic diagnoses, which is in line with our study’s results. This association could potentially be explained by the link between AML and women [33]. Regarding age, our study revealed that the malignant group had an older age, consistent with findings by Xi et al. [21] and Lane et al. [35]. However, other studies have reported varying associations between age and malignancy of renal lesions, which could be due to the heterogeneity of enrolled patients among these studies [21, 22, 33–35]. Our study showed that the addition of clinical factors to the radiomics and ITH model improved the overall AUC by approximately 0.01 in the cross validation. This highlights the potential value of integrating clinical information with features extracted from CT scans to enhance the accuracy of renal lesion differentiation.

To explore whether the combined model could enhance the diagnostic accuracy of radiologists, we fused the predicated risk by combined model and CT reported results. Our study demonstrated a significant improvement in the diagnostic accuracy of combination than the interpretation by radiologists. Another study also showed that radiomics signature help radiologists improve diagnostic accuracy [23]. These findings underscore the radiomics and heterogeneity analysis could capture features overlooked by the naked eye, resulting in an improved diagnostic performance in renal lesion differentiation. Our sensitivity analysis also showed that the combined model (AUC = 0.935) had good discriminative power for SRL. Its performance was comparable to that reported by Dai et al., with AUC values of 0.86, 0.80, and 0.87 for the internal, external, and prospective test sets, respectively, where SRL was defined as ≤ 3.0 cm [36].

Our study has several limitations. Firstly, it was conducted at one hospital, and its retrospective design has inherent drawbacks. However, we implemented temporal validation to enhance the study’s credibility, which is a more robust approach for evaluating model performance [37]. Moreover, participants were enrolled from three different sites of our hospital, and CT data was acquired using various scanners and field strengths, facilitating the development of a more generalizable model. Secondly, our study only included patients who underwent partial nephrectomy, which may introduce selection bias and limit the generalizability of our findings. However, our cohort included a significant number of SRLs (1138, 63.4% of lesions ≤ 4.0 cm), which is valuable for distinguish between benign and malignant in SRLs. Thirdly, manual delineation of ROIs was time-consuming and depend heavily on radiologists’ experience, potentially affecting the stability of the radiomic features. Although we addressed this issue by selecting features with ICC greater than 0.85, implementing an automated and precise tumor segmentation method would further improve efficiency and ensure greater stability and consistency.

## Conclusions

In this study, a machine learning model was developed based on intratumoral radiomics, peritumoral radiomics, heterogeneity analysis, and clinical factors to noninvasively classify benign and malignant renal lesions. The model demonstrated good discrimination, outperforming the interpretation of radiologists. Our findings suggest that the combined model could serve as a practical technique to assist radiologists in clinical practice, particularly in the identification of SRL.

## Supplementary Information

Additional file 1: Fig. S1 The comparison of AUC for predicting benign and malignant renal lesions using AutoGluon-Tabular classifier and Lasso Regression algorithm in the validation (**A**) and test (**B**) cohorts. AUC: area under curve. ITH: intratumoral heterogeneity; ITR: intratumor region; ITR_-3 mm_: ITR with 3 mm shrink; ITR_+3 mm_: ITR with 3 mm expansion; ITR_+5 mm_: ITR with 5 mm expansion; PTR_0~+3 mm_: peritumoral regions of 3 mm around the tumors; PTR_0~+5 mm_: peritumoral regions of 5 mm around the tumors; PTR_-3~+3 mm_: peritumoral regions of 6 mm crossing tumor border; PTR_-3~+5 mm_: peritumoral regions of 8 mm crossing tumor border; ITR_AP_: ITR for arterial phase image; ITR_NCP_: ITR for non-contrast phase image; ITR_VP_: ITR for venous phase image.

Additional file 2: Fig. S2 The performance of the CT reported results, combined model, and fusion of the combined model and CT reported results for differentiation of benign from malignant renal lesions in the whole test cohort and in the test cohort with small renal lesion. ROC curves in the whole (**A**) and small renal lesion (**D**) test cohort; Calibration plot of observed vs predicated risk of malignant renal lesions in the whole (**B**) and small renal lesion (**E**) test cohort; Decision curve analysis for predicting malignant renal lesions in the whole (**C**) and small renal lesion (**F**) test cohort.

Additional file 3: Table S1: The AUC for different combinations of features extracted from CT images.

Additional file 4: Table S2: The importance of predictive features used in the radiomics and ITH model, and the combined model.

Additional file 5: Appendix S1, and Appendix S2.

## Data Availability

The data and material are available through the corresponding author.

## Abbreviations

RCC: Renal cell carcinoma
SRL: Small renal lesion
ITH: Intratumoral heterogeneity
AML: Angiomyolipoma
ITR: Intratumor region
ITR_-3__ mm_: ITR with 3 mm shrink
ITR_+3__ mm_: ITR with 3 mm expansion
ITR_+5__ mm_: ITR with 5 mm expansion
PTR_0~+3__ mm_: Peritumoral regions of 3 mm around the tumors
PTR_0~+5__ mm_: Peritumoral regions of 5 mm around the tumors
PTR_-3~+3__ mm_: Peritumoral regions of 6 mm crossing tumor border
PTR_-3~+5__ mm_: Peritumoral regions of 8 mm crossing tumor border
ICC: Intraclass correlation coefficient
ROC: Receiver operating characteristics
AUC: Area under the curve
CI: Confidence interval
SSR: Sum squares of the residuals
