# Supplementary figures and images for: CT-based conventional radiomics and quantification of intratumoral heterogeneity for predicting benign and malignant renal lesions
Source: Cancer Imaging. 2024 Oct 2;24:130. doi: 10.1186/s40644-024-00775-8 (PMC11446113; doi:10.1186/s40644-024-00775-8)

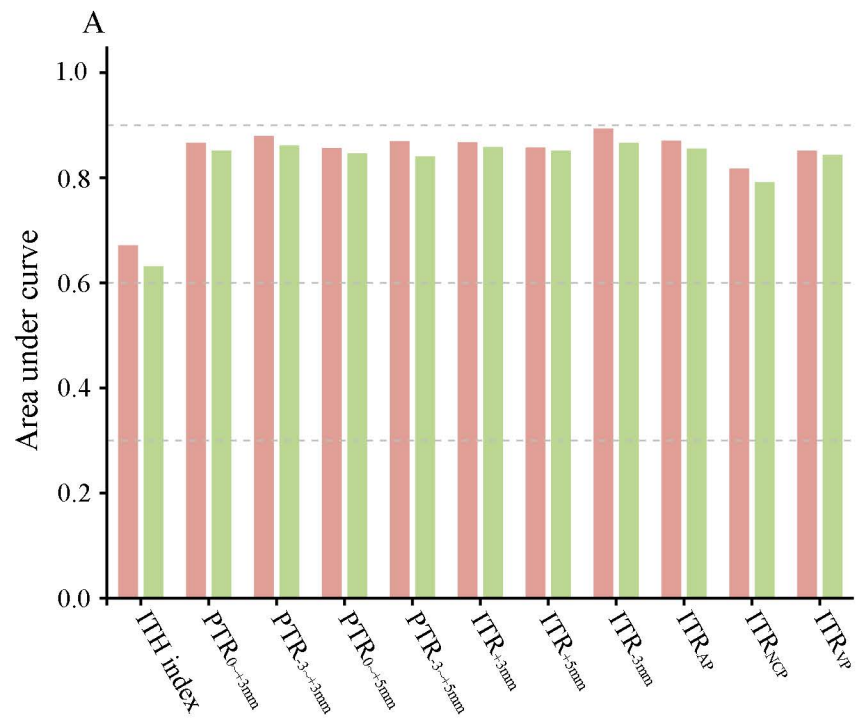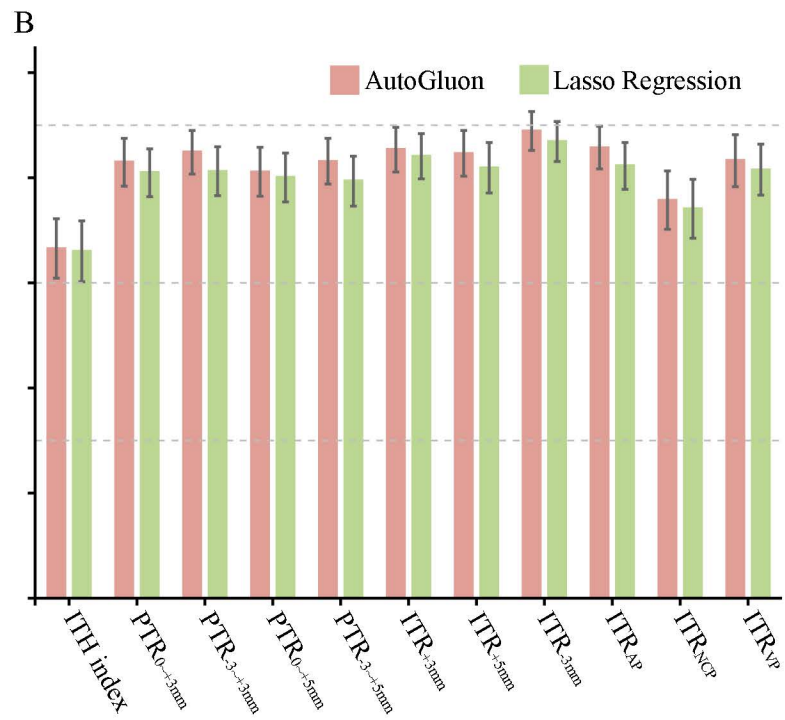

Supplement: Supplementary file 1 — Additional file 1: Fig. S1 The comparison of AUC for predicting benign and malignant renal lesions using AutoGluon-Tabular classifier and Lasso Regression algorithm in the validation (A) and test (B) cohorts. AUC: area under curve. ITH: intratumoral heterogeneity; ITR: intratumor region; ITR-3 mm: ITR with 3 mm shrink; ITR+3 mm: ITR with 3 mm expansion; ITR+5 mm: ITR with 5 mm expansion; PTR0~+3 mm: peritumoral regions of 3 mm around the tumors; PTR0~+5 mm: peritumoral regions of 5 mm around the tumors; PTR-3~+3 mm: peritumoral regions of 6 mm crossing tumor border; PTR-3~+5 mm: peritumoral regions of 8 mm crossing tumor border; ITRAP: ITR for arterial phase image; ITRNCP: ITR for non-contrast phase image; ITRVP: ITR for venous phase image. [file 40644_2024_775_MOESM1_ESM.pdf]

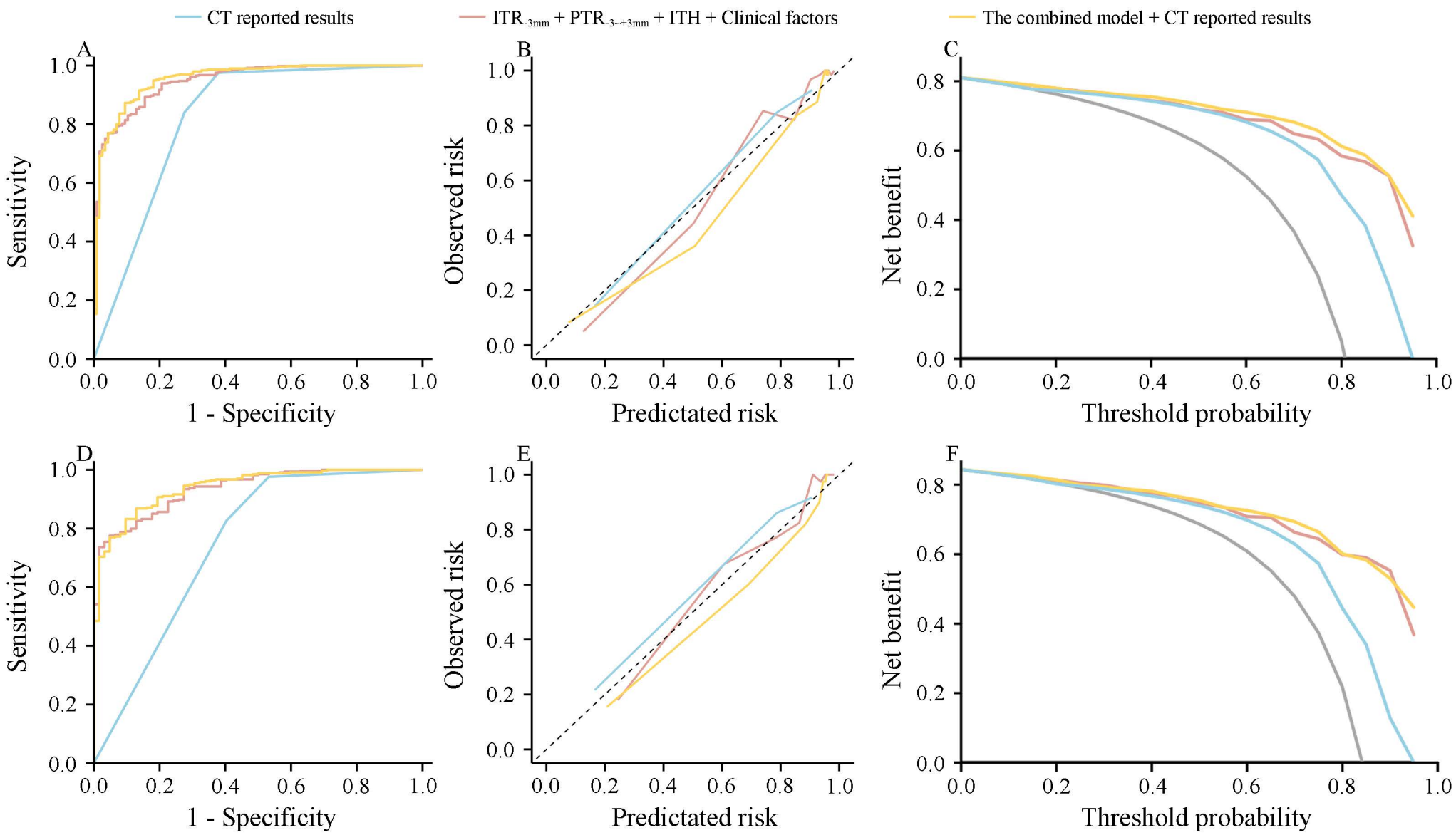

Supplement: Supplementary file 2 — Additional file 2: Fig. S2 The performance of the CT reported results, combined model, and fusion of the combined model and CT reported results for differentiation of benign from malignant renal lesions in the whole test cohort and in the test cohort with small renal lesion. ROC curves in the whole (A) and small renal lesion (D) test cohort; Calibration plot of observed vs predicated risk of malignant renal lesions in the whole (B) and small renal lesion (E) test cohort; Decision curve analysis for predicting malignant renal lesions in the whole (C) and small renal lesion (F) test cohort. [file 40644_2024_775_MOESM2_ESM.pdf]
