# Supplementary material for: CT-based conventional radiomics and quantification of intratumoral heterogeneity for predicting benign and malignant renal lesions
Source: Cancer Imaging. 2024 Oct 2;24:130. doi: 10.1186/s40644-024-00775-8 (PMC11446113; doi:10.1186/s40644-024-00775-8)
Supplement: Supplementary file 3 — Additional file 3: Table S1: The AUC for different combinations of features extracted from CT images. [file 40644_2024_775_MOESM3_ESM.docx]

Table S1: The AUC for different combinations of features extracted from CT images

| **Models** | **Cross validation** | **Test cohorts** |
| --- | --- | --- |
| ITR + PTR_0~+3mm_ | 0.883 | 0.861 (0.821-0.901) |
| ITR + PTR_0~+5mm_ | 0.876 | 0.858 (0.817-0.899) |
| ITR_-3mm_ + PTR_-3~+3mm_ | 0.922 | 0.917 (0.890-0.944) |
| ITR_-3mm_ + PTR_-3~+5mm_ | 0.899 | 0.886 (0.851-0.922) |
| ITR_-3mm_ + PTR_-3~+3mm_ + ITR_NCP_ + ITR_VP_* | 0.921 | 0.909 (0.881-0.937) |
| ITR_-3mm_ + PTR_-3~+3mm_ + ITH | 0.927 | 0.929 (0.904-0.955) |
| ITR_-3mm_ + PTR_-3~+3mm_ + ITH + Clinical factors | 0.939 | 0.946 (0.925-0.968) |

Note: ITR = intratumoral region; PTR_0~+3mm_ = peritumoral region of 3mm around tumor; PTR_0~+5mm_ = peritumoral region of 5mm around tumor; ITR_-3mm_ = ITR with 3 mm shrink; PTR_-3~+3mm_ = peritumoral region of 6mm crossing tumor border; PTR_-3~+5mm_ = peritumoral region of 8mm crossing tumor border; ITR_NCP_ = ITR for non-contrast phase image; ITR_VP_ = ITR for venous phase image; ITH: Intratumoral heterogeneity; * indicated that the analysis was conducted on 1,395 cases, each of which had arterial, non-contrast, and venous phase images.
