# Supplementary material for: CT-based conventional radiomics and quantification of intratumoral heterogeneity for predicting benign and malignant renal lesions
Source: Cancer Imaging. 2024 Oct 2;24:130. doi: 10.1186/s40644-024-00775-8 (PMC11446113; doi:10.1186/s40644-024-00775-8)
Supplement: Supplementary file 4 — Additional file 4: Table S2: The importance of predictive features used in the radiomics and ITH model, and the combined model. [file 40644_2024_775_MOESM4_ESM.docx]

Table S2: The importance of predictive features used in the radiomics and ITH model, and the combined model

| **Features** | **Category** | **Radiomics and ITH model** | |  | **The combined model** | |
| --- | --- | --- | --- | --- | --- | --- |
|  |  | **importance** | **P value** |  | **importance** | **P value** |
| gender | Clinical factors | NA | NA |  | 0.0380 | 0.0001 |
| lbp.3D.m1_firstorder_Mean | ITR_-3mm_ | 0.0166 | 0.0028 |  | 0.0082 | 0.0112 |
| log.sigma.3.mm.3D_glrlm_ShortRunLowGrayLevelEmphasis | ITR_-3mm_ | 0.0061 | 0.0294 |  | 0.0065 | 0.0068 |
| wavelet.LLL_firstorder_RobustMeanAbsoluteDeviation | ITR_-3mm_ | 0.0080 | 0.0038 |  | 0.0060 | 0.0082 |
| original_shape_Elongation | PTR_-3~3mm_ | 0.0055 | 0.0004 |  | 0.0056 | 0.0002 |
| log.sigma.3.mm.3D_firstorder_Maximum | ITR_-3mm_ | 0.0071 | 0.0000 |  | 0.0051 | 0.0008 |
| wavelet.LLL_firstorder_InterquartileRange | ITR_-3mm_ | 0.0043 | 0.0227 |  | 0.0049 | 0.008 |
| log.sigma.3.mm.3D_firstorder_Mean | ITR_-3mm_ | 0.0032 | 0.0216 |  | 0.0049 | 0.0127 |
| log.sigma.5.mm.3D_firstorder_Mean | ITR_-3mm_ | 0.0049 | 0.0016 |  | 0.0048 | 0.0104 |
| log.sigma.3.mm.3D_glszm_LowGrayLevelZoneEmphasis | ITR_-3mm_ | 0.0045 | 0.0046 |  | 0.0044 | 0.003 |
| square_glrlm_LongRunLowGrayLevelEmphasis | PTR_-3~3mm_ | 0.0099 | 0.0007 |  | 0.0043 | 0.0094 |
| wavelet.HHL_glszm_ZoneEntropy | PTR_-3~3mm_ | 0.0037 | 0.0098 |  | 0.0043 | 0.0038 |
| original_shape_Flatness | ITR_-3mm_ | 0.0052 | 0.0002 |  | 0.0041 | 0.0001 |
| age | Clinical factors | NA | NA |  | 0.0040 | 0.001 |
| original_shape_Sphericity | ITR_-3mm_ | 0.0031 | 0.0480 |  | 0.0039 | 0.0144 |
| logarithm_firstorder_Skewness | ITR_-3mm_ | 0.0054 | 0.0004 |  | 0.0038 | 0.0006 |
| log.sigma.5.mm.3D_firstorder_90Percentile | PTR_-3~3mm_ | 0.0048 | 0.0085 |  | 0.0035 | 0.0189 |
| wavelet.LLL_ngtdm_Contrast | PTR_-3~3mm_ | 0.0039 | 0.0022 |  | 0.0035 | 0.0066 |
| wavelet.LLH_firstorder_RootMeanSquared | PTR_-3~3mm_ | 0.0047 | 0.0089 |  | 0.0033 | 0.0126 |
| square_firstorder_Skewness | PTR_-3~3mm_ | 0.0040 | 0.0023 |  | 0.0032 | <0.0001 |
| original_glrlm_LongRunHighGrayLevelEmphasis | PTR_-3~3mm_ | 0.0044 | 0.0038 |  | 0.0032 | 0.0077 |
| logarithm_firstorder_Kurtosis | ITR_-3mm_ | 0.0035 | 0.0001 |  | 0.0031 | 0.0008 |
| log.sigma.5.mm.3D_glcm_JointEntropy | ITR_-3mm_ | 0.0056 | 0.0009 |  | 0.0031 | 0.0045 |
| wavelet.HLL_glszm_LargeAreaHighGrayLevelEmphasis | PTR_-3~3mm_ | 0.0046 | 0.0314 |  | 0.0028 | 0.0184 |
| log.sigma.5.mm.3D_glszm_LowGrayLevelZoneEmphasis | ITR_-3mm_ | 0.0041 | 0.0119 |  | 0.0028 | 0.032 |
| square_firstorder_InterquartileRange | PTR_-3~3mm_ | 0.0018 | 0.0238 |  | 0.0026 | 0.0004 |
| original_shape_Flatness | PTR_-3~3mm_ | 0.0034 | 0.0035 |  | 0.0025 | 0.0006 |
| log.sigma.5.mm.3D_firstorder_Mean | PTR_-3~3mm_ | 0.0020 | 0.0049 |  | 0.0024 | 0.012 |
| wavelet.HLL_gldm_LargeDependenceHighGrayLevelEmphasis | PTR_-3~3mm_ | 0.0014 | 0.0015 |  | 0.0023 | 0.0067 |
| wavelet.LLL_gldm_LowGrayLevelEmphasis | ITR_-3mm_ | 0.0028 | 0.0005 |  | 0.0022 | 0.0053 |
| gradient_glszm_GrayLevelVariance | PTR_-3~3mm_ | 0.0031 | 0.0002 |  | 0.0021 | 0.0064 |
| wavelet.LHL_glcm_Imc2 | PTR_-3~3mm_ | 0.0022 | 0.0032 |  | 0.0021 | 0.0003 |
| original_firstorder_Median | ITH | 0.0018 | 0.0215 |  | 0.0019 | 0.0074 |
| log.sigma.5.mm.3D_firstorder_Entropy | ITR_-3mm_ | 0.0017 | 0.0511 |  | 0.0019 | 0.0018 |
| log.sigma.5.mm.3D_glcm_SumEntropy | ITR_-3mm_ | 0.0012 | 0.0354 |  | 0.0019 | 0.0044 |
| exponential_firstorder_Skewness | PTR_-3~3mm_ | 0.0019 | 0.0014 |  | 0.0018 | 0.0001 |
| log.sigma.5.mm.3D_firstorder_Maximum | ITR_-3mm_ | 0.0027 | 0.0217 |  | 0.0018 | 0.0103 |
| wavelet.LHL_glcm_MCC | PTR_-3~3mm_ | 0.0034 | 0.0015 |  | 0.0016 | 0.005 |
| log.sigma.5.mm.3D_firstorder_RootMeanSquared | PTR_-3~3mm_ | 0.0035 | 0.0054 |  | 0.0014 | 0.025 |
| wavelet.LHL_firstorder_Range | PTR_-3~3mm_ | 0.0026 | 0.0046 |  | NA | NA |
| exponential_glszm_HighGrayLevelZoneEmphasis | PTR_-3~3mm_ | 0.0016 | 0.0129 |  | NA | NA |
| original_firstorder_Kurtosis | ITR_-3mm_ | 0.0013 | 0.0011 |  | NA | NA |
| original_firstorder_Skewness | PTR_-3~3mm_ | 0.0010 | 0.0507 |  | NA | NA |
| lbp.3D.k_firstorder_Range | PTR_-3~3mm_ | 0.0009 | 0.0058 |  | NA | NA |
| log.sigma.5.mm.3D_firstorder_90Percentile | ITR_-3mm_ | 0.0007 | 0.0328 |  | NA | NA |

Note: ITR_-3mm_ = intratumoral region (ITR) with 3 mm shrink; PTR_-3~3mm_ = peritumoral region of 6mm crossing tumor border; ITH = Intratumoral heterogeneity; NA = not applicable.
