# Supplementary material for: CT-based conventional radiomics and quantification of intratumoral heterogeneity for predicting benign and malignant renal lesions
Source: Cancer Imaging. 2024 Oct 2;24:130. doi: 10.1186/s40644-024-00775-8 (PMC11446113; doi:10.1186/s40644-024-00775-8)
Supplement: Supplementary file 5 — Additional file 5: Appendix S1, and Appendix S2. [file 40644_2024_775_MOESM5_ESM.docx]

**Supplementary Material**

**Appendix S1**

All CT scans were performed with one of the following scanners: Phillips 256 iCT, GE Discovery CT750 HD scanner and GE Revolution CT. The CT scanning parameters were as follows: tube voltage, 120kVp; tube current: 200-350 mA; rotation time, 0.5 s; contrast agent type, Omnipaque, GE Healthcare, USA; contrast agent concentration, 350 mg/mL; contrast agent dosage, 1.5 ml/kg body weight; contrast agent infused rate, 3.0 mL/s; arterial phase interval time, 30 s after injection of contrast agent; venous phase interval time, 60-70 s after injection of contrast agent; field of view, 500×500 mm; matrix, 512×512; reconstruction thickness, 0.625–5.0 mm.

**Appendix S2**

CT images were loaded into ITK-SNAP (Version 3.8.0), anonymized and saved in NiFTI format. The arterial phase, providing the clearest tumor delineation, served as the reference target for subsequent co-registration with other phases. To obtain voxel-level segmentation labels, three trained annotators, supervised by a radiologist with 10 years of experience in abdominal radiology, used ITK-SANP to delineate the lesion contours slice-by-slice on axial images of arterial phase, and these annotations were thoroughly checked and refined by the experienced radiologist. To assess interobserver and intraobserver agreement, the same three annotators repeated performed segmentations on the 30 randomly selected CT images within one month.

After the generation of the intratumoral regions (ITRs), the arterial phase images were resampled to a voxel size of 1×1×1 mm using stickBSpline interpolator. Subsequently, ITR with 3 mm shrink (ITR_-3mm_) was created by eroding the original ITR, and ITR with 3 (ITR_+3mm_) and 5 mm (ITR_+5mm_) expansion was obtained by dilating the original ITR using scipy.ndimage module (Version: 1.11.3). Peritumoral regions (PTRs) of 3 (PTR_0~+3mm_) and 5 mm (PTR_0~+5mm_) around the tumors were obtained by subtracting ITR from ITR_+3mm_ and ITR_+5mm_, and 6 (PTR_-3~+3mm_) and 8 mm (PTR_-3~+5mm_) crossing tumor border was obtained by subtracting ITR_-3mm_ from ITR_+3mm_ and ITR_+5mm_. Eight regions of interest (ROIs) were obtained for each lesion, namely, ITR, ITR_-3mm_, ITR_+3mm_, ITR_+5mm_, PTR_0~+3mm_, PTR_0~+5mm_, PTR_-3~+3mm_, PTR_-3~+5mm_, to extract radiomic features from arterial phase images.

The ITKElastix package (Version 0.17.0) was utilized to perform co-registration of non-contrast and venous phase images to the arterial phase images. The registration process involved a combination of rigid, affine, and B-spline transformations to ensure spatial consistency across various phases.

Before feature extraction, image preprocessing, including normalization, and discretization steps, was applied to all the data. Normalization was performed using the z-score method, and voxel intensities were discretized by employing a bin-width of 25 Hounsfield units. For arterial phase images, all eight ROIs were used for radiomic feature extraction; for non-contrast and venous phase images, only ITR were used to extract radiomic features. Finally, each renal lesion yielded a total of 17,810 (1,781×10) radiomic features.
